# Motion as a Cause of Aggravation*This essay of Dr. Bœnninghausen was published by Dr. Dunham twenty years ago in the *American Homœopathic Review*. To most of our readers it is probably new, to all it will be interesting and instructive, as it gives many useful therapeutic hints and also teaches us the great minuteness necessary for a correct homoeopathic prescription.—Editor

**Published:** 1882-12

**Authors:** C. Von Bœnninghausen


### MOTION AS A CAUSE OF AGGRAVATION.*

DR. C. VON BŒNNINGHAUSEN.

The occurrence or aggravation of (internal as well as external) symptoms by *motion* of the whole body or only of the part affected is without doubt known in a general way to every homœopathist. No one would, for example, give Bryonia in a so-called nervous fever if patient kept constantly tossing about and could find no rest on account of pains in the limbs which were relieved only by motion; neither would he give Rhus in a disease going by the same pathological name if every motion, however trivial, aggravated to an intolerable degree the pains in the limbs and if the pains were only relieved by repose.

It would, however, be a mistake to suppose that with these general designations, *motion* and *rest*, the subject is exhausted. In this very particular it is most evident, as in like manner with regard to many other influencing circumstances, with what sharpness and precision the examination of the patient must be made if we would select the remedy with as much certainty as that with which we desire to

*This essay of Dr. Bœnninghausen was published by Dr. Dunham twenty years ago in the *American Homœopathic Review*. To most of our readers it is probably new, to all it will be interesting and instructive, as it gives many useful therapeutic hints and also teaches us the great minuteness necessary for a correct homœopathic prescription.—EDITOR.

cure the disease. The following pages are devoted to a closer consideration of motion and rest such as has been alluded to. They are the fruit of careful experiment and observation for many years, and as such I venture to commend them to my younger colleagues.

When a patient in reply to a question simply says, "Motion aggravates," this may be understood in a threefold sense. The aggravation may take place (1) on *beginning to move*, (2) *during continued motion*, or (3) immediately *after moving*. These are clearly important distinctions which are wont to be predicated of motion and to be specified as legitimate sequences of it, but *each* of which has special relation to its own peculiar group of remedies.

When, for example, the aggravation takes place only at the *beginning* of motion and diminishes gradually as the motion is *continued*, then Caps., Carbo v., Caust., Con., Euphor., Ferr., Fluor. ac., Lyc., Puls., Rhus, Sabad., Samb., and Silicea are most frequently indicated. When, on the other hand, the aggravation occurs *during* motion and is increased by *continuance* of the motion, our first choice will be Bell., Bry., Cocc., Colch., Led., Nux v., etc. When, however, the aggravation occurs immediately *after* motion, that is, in the period of repose which immediately follows motion, still other remedies are to be preferred, such as Agar., Anac., Ars., Cann., Hyos., Kali c., Puls., Rhus, Ruta, Sepia, Spong., Stann., Stram., Valer., Zinc.

Important as it is to notice the above distinctions, yet in many cases even this is not sufficient. There exist in reference to motion and rest still other points which require equally to be observed, inasmuch as they correspond, like the foregoing, to the *individual* genius of the different remedies.

First, it makes an important difference whether the motion is violent, involving much bodily exertion, in which case, while observing the distinctions previously defined, preference is to be given to Acon., Arn., Ars., Bry., Calc., Cann., Lyc., Nux v., Rhus, Ruta, Sil., Sulph.

If, further, there has been, in conjunction with the motion, considerable *overheating*, they will fall especially among Acon., Ant. c., Bell., Bry., Camph., Carbo v., Dig., Kali c., Opium, Phos., Sep., Sil., Thuja, Zinc. What might be said of the same character respecting taking cold (simultaneously with and immediately after motion) either in the entire body or in isolated parts, whether from getting wet through or from some other causes—circumstances which might sometimes require the selection of still other remedies—this we must omit for lack of space.

It is necessary, however, to state, briefly, that the *kind* and *manner* of the motion likewise furnish their peculiar indications. Thus, for aggravation from assuming the erect position, we have Acon., Bell., Bry., Ign., Nux v., Op., Rhus, Staph., Sulph.; while aggravation from stooping: Alum., Amm c., Arn., Calc., Lach., Mang., Sep., Spig., Thuja, Valer. Although the desired favorable result may be wrought by still other remedies, especially by such as produce alternate effects when they correspond homœopathically in respect of symptoms.

The same is true of aggravation on rising from a sitting posture, which requires chiefly Acon., Apis, Caps., Con., Fluor. ac., Lyc., Phos., Puls., Rhus, Spig.; aggravation on rising from the recumbent posture (from the bed) calls for Apis, Bry., Carbo v., Con., Lach., Sul. ac.

As a matter of course, under these heads the beginning of motion is involved in assuming the upright posture, and in rising (from the bed) is involved also the aggravation of symptoms after sleep, and thus still other remedies may come under consideration.

Furthermore, it is to be observed whether the aggravation of symptoms takes place during or after the rising from a sitting or recumbent posture, because in these cases, as has been before remarked of motion in general, different remedies compete for a preference.

The kind of motions must be carefully noticed. Aggravation from extending the part affected is a verified indication for Alum., Calc., Coloc., Rhus, Sep., Staph., Sulph., Thuja; and aggravation from *flexing* or *turning* it, for Amm. m., Cicuta, Ign., Kali c., Lyc., Nux v., Puls., Spig., Spongia. The direction in which the motion of flexion is executed makes an important difference. If outward, Caps. and Caust. are preferable; if inward, Ign. and Staph.; if backward, Calc., Kali c., Puls., Sepia, and Sulph.; if sidewise, Bell. and Natr. m.; if forward, Coff. and Thuja; or, finally, if the part be retained in the flexed position, Hyos., Spong., and Valer. Under the head of extension belong also stretching and twisting, for which, likewise, certain remedies are especially indicated: Amm. c., Ran. b., and Rhus; as well as drawing up a limb, which frequently indicates Ant. t., Rhus, and Secale.

Under this general head come also aggravations from lifting the affected limb, for which Arn., Baryta, Bell., Ferr., Kali c., Led., Rhus, and Silicea are indicated; and in particular from straining, for which Arn., Bor., Bry., Calc., Cocc., Graph., Ign., Lyc., Natr. c., Nux v., Ph. ac., Rhus, Sep., Sil., and Sulph. stand in the first rank as remedies.

If *walking* in general is to be included under the head of *motion*, then the distinctions above specified of motion will apply to walking, and aggravations occurring on beginning to walk will have a corresponding therapeutic value. But there are certain additional varieties which furnish special indications by reason of their connection with certain accessory circumstances; for example, walking in the open air gives rise to aggravation of a great number of symptoms, and hence serves as an indication for a large number of remedies, but especially for the following: Anac., Bell., Carbo v., Cocc., Colch., Con., Fluor. ac., Hepar, Nux v., Ph. ac., Selen., Spig. and Sulph.

But even this is far from exhausting our therapeutic store. The additional question arises whether this aggravation on walking in the open air occurs in a *damp atmosphere* or *in rainy weather*, in which case Amm. c., Calc., Colch., Dulc., Fluor. ac., Lach., Lyc., Nux m., Rhus, Sulph. or Verat. are usually indicated; or whether it takes place in *dry weather*, in which event Asar., Bell., Bry., Caust., Hepar., Nux v. and Puls. are especially indicated.

Moreover, special indications are furnished—by aggravation from *exposure to the hot sun*—for Ant. c., Bell., Bry., Lach., Natr. c., Puls., Selen., Valer.; from exposure to *air just before a thunderstorm*, for Agar., Natr. c., Phos., Rhod., and Sil.; from exposure to *snowy air*, for Colc., Con., Lyc., Phos., Ph. ac., Puls., Rhus, Sep., Sil., Sulph.; and from exposure to *fog*, for Bry., Cham., China, Mang., Nux m., Rhod., Rhus, Sep., Sulph., Verat. Under this head belongs also *walking in the wind*, and Ars., Asar., Bell., Calc., Cham., China, Euphra., Graph., Lach., Lyc., Nux v., Phos., Puls., Rhus, Spig., and Thuja are especially indicated when aggravation occurs from walking in a strong wind.

Besides the preceding conditions which exert an influence in a therapeutic point of view upon the motion of walking, there are several others which sometimes accompany isolated symptoms and furnish indications that are all the more useful, because in such cases leading concomitants are often altogether lacking. Such are, for example, under *vertigo*, the aggravation by walking over a *narrow bridge*, which indicates Baryta, Ferr., and Sulph.; or upon or over *water*, which indicates Angust., Ferr., and Sulph. The same is true of pains in the soles of the feet, aggravated by *walking upon a hard floor* or upon a cement walk, a condition which calls for Ant. c., Ars., Con. and Hepar.

But *motion upward or downward* requires also very particular consideration. For *motion upward* (ascending, going up stairs), among many other remedies, Arn., Ars., Bry., Cupr., Nux v., Senega, Sep., Spig. and Spong. are most prominent; while for *downward motion* (descending, going down stairs), Arg., Con., Ferr., Lyc., Rhod., Ruta, Sabina and Verat. have in many cases proved to be indicated.

*Driving and riding* must be included among the varieties of motion. Various symptoms are induced or aggravated by driving in a wagon which generally find their remedy among Ars., Bry., Cocc., Colch., Hepar, Hyos., Ign., Lach., Nux m., Op., Petro., Rhus, Selen., Sep., Sil. and Sulph. On the other hand, the remedies for *sea-sickness* from motion in a ship are pretty much confined to Ars., Cocc., Colch., Ferr., Hyos., Op., Petro., Sep., Sil. and Tabac., although the *motion of rocking*—which seems so nearly related to the above—corresponds only to Borax and Carbo veg. It may here be mentioned as something remarkable that some symptoms are *relieved by driving in a wagon*, and in such cases Ars., Graph., Nitr. ac. or Phos. are most likely to be indicated.

As regards riding (on horseback), the totality of the symptoms in those persons with whom this exercise does not agree will generally be found to be of such a character that Graph., Natr. c., Sep., Spig. or Sul. ac. are among the remedies best indicated. In this connection, as above, we note a singular circumstance, viz.: that cases of exceedingly painful, inflamed, and protruding hæmorrhoids sometimes present themselves in which, contrary to all analogy and to all reasonable expectation, riding (on horseback) affords the greatest relief. In such cases as these a single very small dose of a high potency of Kali carb. is generally sufficient to cure the disease rapidly and permanently.*

Just as *change of position* may, through the aggravation of symptoms, furnish a useful indication for several remedies—most strik-

---

* For an example of this, see case reported by Dr. McNeil, in October (1882) issue of this journal.—EDITOR.

ingly for Caps., Carbo v., Con., Euphor., Lach., Lyc., Phos., Puls. and Samb.—so it may also happen that it alleviates the symptoms. This affords a very characteristic indication for Cham., Ign., Ph. ac., Valer. or Zinc.

*Turning over in bed* is also a motion which produces more or less aggravation under several remedies, and may, therefore, serve as an indication. It is most marked under Acon., Ars., Borax, Bry., Cann., Caps., Carbo. v., Con., Ferr., Hepar, Lyc., Natr. m., Nux v., Puls., Rhus, Sil., Staph. and Sulph. Closely related to this is the motion of *looking around*, although as regards aggravation it is as yet noted only of Calc., Cicuta, Con., Ipec. and Kali carb.

In addition to the above-named varieties of motion there are many others, which, however, we may pass by here, the rather because they affect often only isolated portions of the body, a fact which constitutes in itself something of an individual characteristic, and which, therefore, gives the motion associated with it a somewhat subordinate rank. Among the number are, for example, *respiration, inspiration,* as well as *expiration,* swallowing—whether only of *saliva,* or *empty* swallowing, or swallowing of *food* or *drink; sneezing, yawning, coughing, speaking, writing,* etc. Respecting all of these conditions, in so far as they exercise an influence upon the aggravation or amelioration of symptoms, our materia medica pura contains a large number of observations which were first obtained by provings upon the healthy and then verified by administration to the sick. They have, therefore, sustained a double test, *a priori* and *a posteriori,* and they deserve just as much consideration, in the search for the most complete and perfect *simile,* as any other symptoms that have been discovered and verified in the same way. If, at the present day, this consideration is not commonly accorded—if, indeed, the attention is chiefly fixed on the pathological, the general symptoms, while the concomitant symptoms—which, for the most part, are very characteristic—are correspondingly neglected—assuredly such laxity and incompleteness in the application of our fundamental principle are utterly inexcusable, and it is not to be wondered at that even "experiments on the sick," to their great injury, should be ever on the increase, while *pure experiment* is growing less and less frequent.

